# The Evolving Kidney Donor Pool Shaping Outcomes in Graft Survival

**DOI:** 10.1155/joot/3352344

**Published:** 2026-02-24

**Authors:** Kaufman Daniel M., Perkins James D., Bakthavatsalam Ramasamy, Leca Nicolae, Sibulesky Lena

**Affiliations:** ^1^ Department of Surgery, Division of Transplant Surgery, St. Vincent Hospital, Indianapolis, Indiana, USA, stvincenthospital.com; ^2^ Department of Surgery, Division of Transplant Surgery, University of Washington, Seattle, Washington, USA, washington.edu; ^3^ Clinical and Bio-Analytics Transplant Laboratory (CBATL), University of Washington, Seattle, Washington, USA, washington.edu; ^4^ Department of Medicine, Division of Nephrology, University of Washington Medical Center, Seattle, Washington, USA, uwmedicine.org

**Keywords:** DCD kidney transplant, donor risk, drug overdose deaths, KDPI, kidney donors, kidney graft survival, kidney transplant

## Abstract

**Background:**

The composition of the U.S. deceased kidney donor pool is undergoing a major shift. While the past decade saw an increase in younger, overdose‐death donors associated with favorable transplant outcomes, recent years have marked a decline in these donors. Concurrently, there has been a rise in higher‐risk donor characteristics—namely, older age, more comorbidities, such as hypertension and diabetes, and increased reliance on donation after circulatory death (DCD). These trends may significantly affect transplant outcomes.

**Methods:**

We analyzed data from the Organ Procurement and Transplantation Network (OPTN) on 101,550 deceased kidney donors (2018–Q1 2025) and 108,611 single kidney transplants (2018–mid‐2024). Donor trends were assessed using segmented regression analysis. Graft survival was evaluated using Kaplan–Meier survival curves and multivariable Cox proportional hazards models, adjusting for donor, recipient, and transplant characteristics.

**Results:**

Overdose‐death donors declined from 16.7% in 2022 to 10.5% by Q1 2025. Simultaneously, DCD donors rose to nearly 50% of all deceased donors. The proportion of older donors and high‐KDPI kidneys also increased. Kaplan–Meier analysis showed a decrease in unadjusted death‐censored graft survival in 2024 compared to 2018–2022 (97.0% vs. 97.6%, *p* < 0.001). In adjusted Cox models, donor factors—DCD status, older age, hypertension, diabetes, and prolonged cold ischemia—were independently associated with graft loss.

**Conclusions:**

The U.S. kidney donor pool is shifting toward higher‐risk profiles, with early signs of declining graft survival. Strategies to optimize organ preservation and allocation will be essential to maintain transplant outcomes amid these changing donor trends.

## 1. Introduction

The landscape of the deceased donor kidney pool in the United States has changed dramatically over the past two decades. Historically, donors were often young individuals who died from traumatic injuries, providing organs of relatively high quality. In the 2000s and 2010s, the opioid epidemic gave rise to a new donor profile: donors who died from overdoses. Despite initial concerns about infectious disease transmission, improvements in screening and antiviral therapies allowed these organs to be used safely, and outcomes were often favorable given the younger age and lower comorbidity burden of these donors [1, 2].

In recent years, however, the donor landscape has shifted again. National overdose‐related deaths peaked in 2022 but have since declined substantially [3]. At the same time, the overall number of deceased donors has continued to rise, marked instead by growth in older donors, those with comorbid conditions, such as hypertension and diabetes, and increase in donation after circulatory death (DCD) [4]. These changes have led to greater reliance on high Kidney Donor Profile Index (KDPI) kidneys and have raised concerns about the long‐term sustainability of transplant outcomes.

While individual risk factors, such as older donor age, DCD, and elevated KDPI, are well‐established predictors of graft loss [5, 6], the cumulative impact of multiple simultaneous shifts in the donor pool on recipient outcomes has not been fully evaluated.

The aim of this study was to provide a comprehensive analysis of recent trends in the U.S. kidney donor pool and their association with early graft outcomes. Specifically, we evaluated temporal changes in donor demographics and risk profiles from 2018 through early 2025, and we examined whether these shifts correspond with measurable changes in kidney utilization and death‐censored graft survival. By reframing the current donor landscape as a transition toward higher risk, we highlight the need for early recognition and adaptation to preserve kidney transplant success in the years ahead.

## 2. Methods

Data for this study were obtained from the Organ Procurement and Transplantation Network (OPTN) and released in April 2025. The United Network for Organ Sharing (UNOS), as the contractor for OPTN, provided access to de‐identified national registry datasets. Because these data are publicly available and anonymized, the study was deemed exempt from human subjects review and institutional ethics approval.

Two datasets were analyzed. The first included all deceased kidney donors in the United States between January 1, 2018, and March 31, 2025 (*n* = 101,550). Donor characteristics evaluated included age, mechanism of death, comorbidities, such as hypertension and diabetes mellitus, Kidney Donor Profile Index (KDPI), DCD status, hepatitis C serology, and preservation method (pump vs. static cold storage). The second dataset included all adult recipients (≥ 18 years) of single‐organ deceased donor kidney transplants performed between January 1, 2018, and June 30, 2024, with follow‐up through March 31, 2025 (*n* = 108,611). Recipients of living donor, dual, or multiorgan transplants were excluded from the analysis.

The primary outcome of interest was 180‐day death‐censored graft survival. Secondary outcomes included delayed graft function (DGF), primary nonfunction (PNF), and serum creatinine at six months.

To evaluate changes in donor risk over time, temporal shifts in donor characteristics (age groups, KDPI ≥ 86%, overdose‐related deaths, and DCD) were assessed by calendar quarter using segmented linear regression. Breakpoints were determined empirically, with Q1 2023 identified as the most consistent inflection point. Kidney utilization was assessed by examining the proportion of recovered, transplanted, and discarded kidneys annually between 2018 and 2024 [4, 7].

For survival analysis, Kaplan–Meier methods were used to estimate 180‐day death‐censored graft survival, and survival across eras (2018–2022, 2023, and 2024) was compared using log‐rank testing. Univariable and multivariable Cox proportional hazards models were constructed to evaluate the association of transplant year and other covariates with graft failure. Covariates included donor age, comorbidities, KDPI, DCD, mechanism of death, cold ischemia time, and preservation method; recipient factors, such as age, diabetes mellitus, dialysis duration, prior kidney transplant, peripheral vascular disease, body weight, and calculated panel reactive antibody (cPRA); and transplant characteristics including HLA mismatches, induction immunosuppressive therapy, and organ sharing region.

Multicollinearity was assessed using variance inflation factors, and variables with high collinearity were removed or modified. The proportional hazards assumption was tested using Schoenfeld residuals. Missing data were minimal. For variables with less than 1% missingness, the median was used for continuous variables and the mode for categorical variables. For donor weight and cold ischemia time, regression‐based imputation was applied using related covariates.

All analyses were performed using JMP Pro Version 17.0 (SAS Institute, Cary, NC) and Python Version 3.13 (pandas, lifelines, and statsmodels). A two‐sided *p* value < 0.05 was considered statistically significant.

## 3. Results

### 3.1. Donor Trends

**FIGURE 1:**
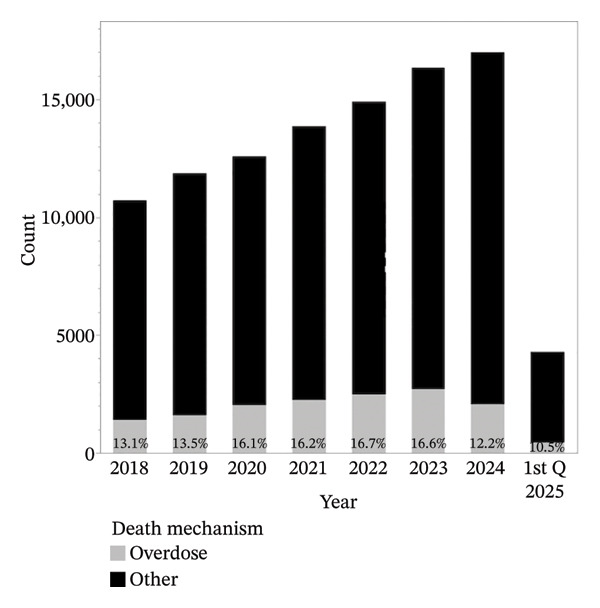
Annual trends in total deceased donors and overdose‐related deaths, 2018–Q1 2025.

Between 2018 and the first quarter of 2025, the total number of deceased kidney donors in the United States increased steadily (Table 1; Figure 1). However, the composition of the donor pool shifted substantially. Overdose‐related deaths, which accounted for 16.7% of donors in 2022, declined sharply to 12.2% in 2024 and 10.5% in Q1 2025. In contrast, DCD rose from 19.9% in 2018 to 42.9% in 2024 and nearly half of all donors by early 2025.

**TABLE 1:** Annual donor characteristics and counts, 2018–Q1 2025.

Characteristic	2018	2019	2020	2021	2022	2023	2024	Q1 2025	*p* value^2^
Number^1^	10,721	11,870	12,588	13,862	14,904	16,336	16,989	4280	
Overdose deaths	1402 (13.1%)	1604 (13.5%)	2031 (16.1%)	2248 (16.2%)	2485 (16.7%)	2714 (16.6%)	2065 (12.2%)	450 (10.5%)	< 0.001
DCD donors	2132 (19.9%)	2718 (22.9%)	3224 (25.6%)	4190 (30.2%)	4777 (32.1%)	5894 (36.1%)	7284 (42.9%)	2060 (48.2%)	< 0.001
Donor age groups									< 0.001
Age 0–54	8098 (75.5%)	8780 (74.0%)	9353 (74.3%)	10,105 (72.9%)	10,694 (71.8%)	11,242 (68.8%)	10,631 (62.6%)	2251 (59.1%)	
Age 55–64	1828 (17.1%)	2235 (18.8%)	2416 (19.2%)	2774 (20.0%)	3070 (20.6%)	3600 (22.0%)	4234 (24.9%)	1105 (25.8%)	
Age ≥ 65	795 (7.4%)	855 (7.2%)	819 (6.5%)	983 (7.1%)	1140 (7.7%)	1494 (9.2%)	2124 (12.5%)	644 (15.1%)	
KDPI 86–100	1160 (10.8%)	1422 (12.0%)	1423 (11.3%)	1820 (13.1%)	2039 (13.7%)	2715 (16.6%)	3825 (22.5%)	1150 (26.9%)	< 0.001
Kidney donor outcome									
Recovered	9867 (92.0%)	11,152 (94.0%)	11,925 (94.7%)	13,215 (95.3%)	14,227 (95.5%)	15,471 (94.7%)	15,937 (93.8%)	3956 (92.4%)	< 0.001
Transplanted	8368 (84.8%)	9385 (84.2%)	9884 (82.9%)	10,624 (80.4%)	11,180 (78.6%)	11,930 (77.1%)	12,127 (76.1%)	3039 (76.8%)	< 0.001
Discarded	1499 (15.2%)	1767 (15.8%)	2041 (17.1%)	2591 (19.6%)	3047 (21.4%)	3541 (22.9%)	3810 (23.9%)	917 (23.2%)	< 0.001

^1^Total number of deceased kidney donors.

^2^ *p* value from Pearson’s chi‐squared test or Fisher’s exact test, as appropriate.

Median donor age also increased throughout the different time periods. The proportion of younger donors (0–54 years) fell from 75.5% in 2018 to 62.6% in 2024, while donors aged 55–64 years rose to 24.9% and those ≥ 65 years to 12.5%. Parallel with these shifts, the proportion of kidneys with KDPI ≥ 86% increased more than twofold, from 10.8% in 2018 to 22.5% in 2024. Kidney utilization reflected these trends: The proportion of recovered kidneys that were transplanted fell from 84.8% in 2018 to 76.1% in 2024, while nonutilization rates rose to nearly one in four by 2024.

### 3.2. Temporal Shifts in Donor Characteristics

To better visualize the evolving composition of the donor pool, segmented linear regression was performed on quarterly data from 2018 through Q1 2025. A consistent inflection point was identified at Q1 2023 across all models. For all analyses, beta coefficients represent the average change in donor characteristics expressed in percentage points per quarter.

Overdose‐related donor deaths increased at a rate of 0.33% points per quarter before Q1 2023 (*p* < 0.001), but this trend reversed sharply after the breakpoint, with a decline of 1.62% points per quarter (*p* < 0.001) (Figure 2(a)). The proportion of donors recovered as DCDs also rose steadily, increasing by 0.60% points per quarter before Q1 2023 and accelerating to 0.80% points per quarter thereafter (*p* < 0.001 for both) (Figure 2(b)). The proportion of donors aged 0–54 years declined slightly before Q1 2023 (*β* = −0.07, *p* = 0.09), but the decrease became more pronounced after the breakpoint (*β* = −1.23, *p* < 0.001) (Figure 2(c)). In concert, the proportion of donors aged 55–64 years showed a statistically significant increase before the breakpoint (*β* = 0.08, *p* = 0.03), with a much steeper increase observed after Q1 2023 (*β* = 0.67, *p* < 0.001) (Figure 2(d)). The proportion of donors aged ≥ 65 years remained stable through early 2023 (*β* = −0.004, *p* = 0.78), but increased significantly thereafter (*β* = 0.56, *p* < 0.001) (Figure 2(e)). In parallel with these demographic and clinical changes, the proportion of high‐risk kidneys, defined by a Kidney Donor Profile Index (KDPI) ≥ 86, rose modestly before Q1 2023 (*β* = 0.05, *p* = 0.02) and then increased sharply (*β* = 0.82, *p* < 0.001) (Figure 2(f)). This shift reflects the combined impact of increasing donor age and DCD use on calculated donor risk.

FIGURE 2Segmented linear regression of quarterly donor pool characteristics, 2018–Q1 2025. (a) Overdose‐related donor deaths. (b) Donation after circulatory death (DCD). (c) Donors aged 0–54. (d) Donors aged 55–64. (e) Donors aged ≥ 65. (f) High‐risk kidneys (KDPI ≥ 86).

**Figure:**
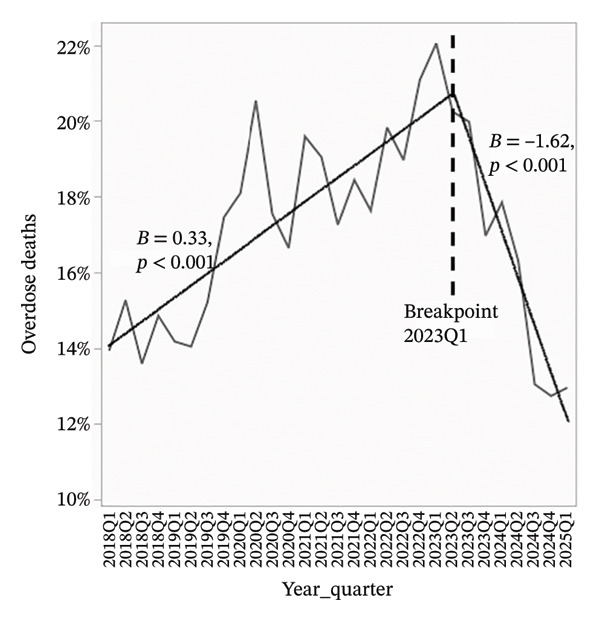
(a)

**Figure:**
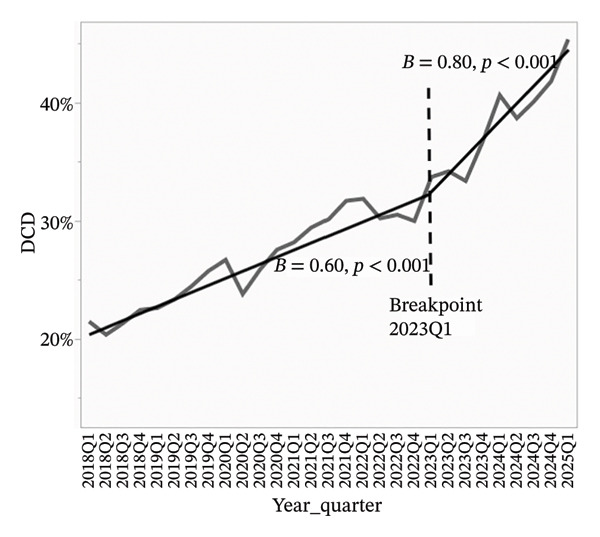
(b)

**Figure:**
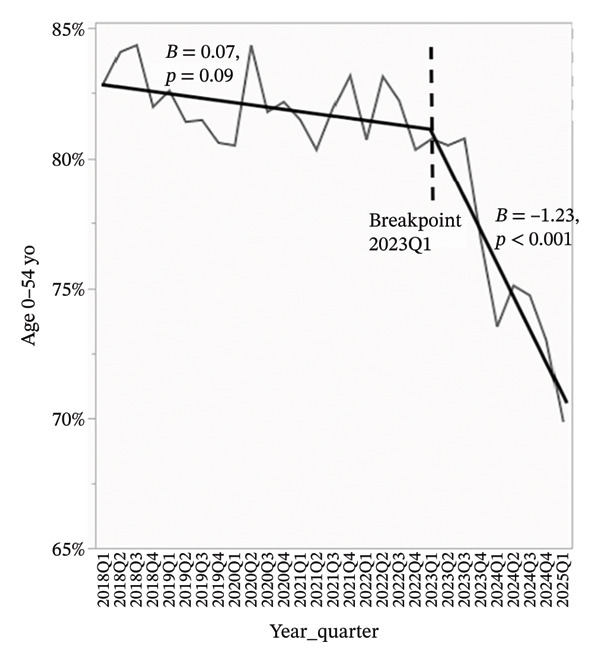
(c)

**Figure:**
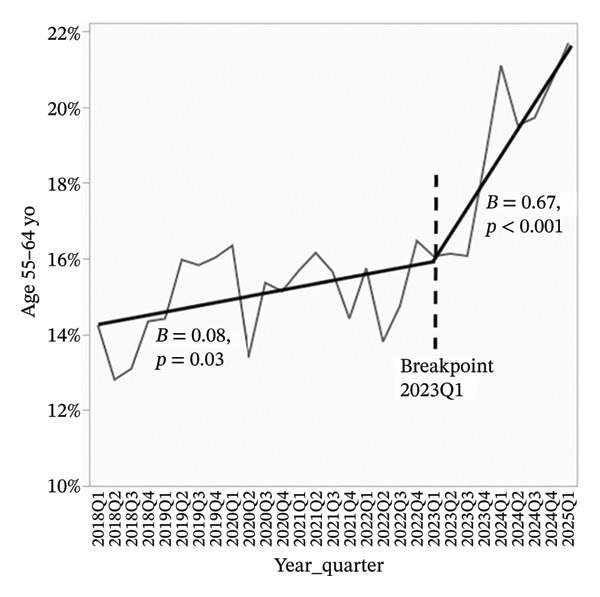
(d)

**Figure:**
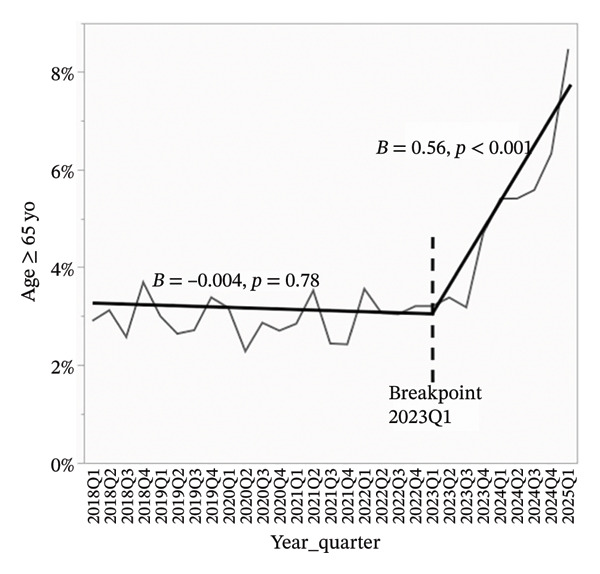
(e)

**Figure:**
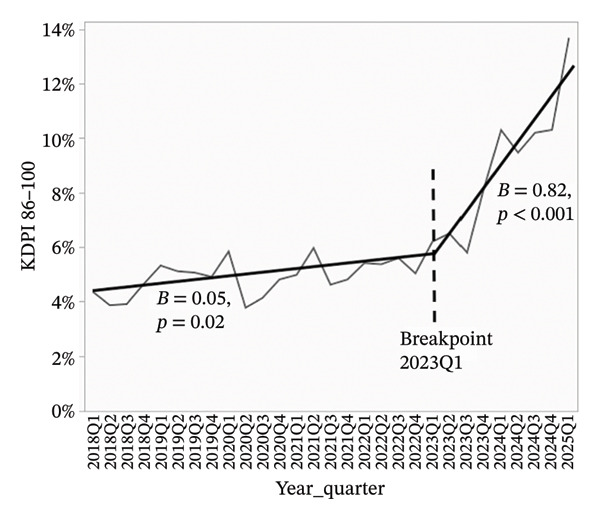
(f)

### 3.3. Transplant Characteristics

Donor and transplant features of kidney recipients reflected these broader national trends (Table 2). In 2024, a smaller proportion of transplants were derived from overdose donors (17.6%) compared with 20.6% in 2023. Donors aged 55–64 years increased to 20.9%, and those ≥ 65 years doubled to 5.1%. Use of DCD kidneys rose from 29.5% in 2018–2022 to 42.0% in 2024. Donor comorbidities also became more prevalent, with hypertension increasing to 36.0% and diabetes mellitus to 11.9%.

**TABLE 2:** Baseline donor, recipient, and transplant characteristics for kidney transplants performed between 2018 and 2022, 2023, and January 1, 2024–June 30, 2024.

Variables	2018–2022	2023	2024^1^	*p* value^2^
Number^3^	79,390	19,245	9976	
Donor				
Single kidney vs dual/en bloc	77,861 (98.1%)	18,878 (98.1%)	9802 (98.3%)	0.45
Death mechanism				< 0.001
Overdose	14,199 (17.9%)	3960 (20.6%)	1762 (17.6%)	
Other	65,191 (82.1%)	15,285 (79.4%)	8214 (82.4%)	
Donor age groups				< 0.001
0–54 yo	64,731 (81.5%)	15,324 (79.6%)	7385 (74.0%)	
55–64 yo	12,462 (15.7%)	3289 (17.1%)	2086 (20.9%)	
≥ 65 yo	2197 (2.8%)	632 (3.3%)	505 (5.1%)	
Race/ethnicity				< 0.001
Asian	1915 (2.4%)	461 (2.4%)	249 (2.5%)	
Black	10,856 (13.7%)	2712 (14.1%)	1297 (13.0%)	
Hispanic	11,962 (15.1%)	2850 (14.8%)	1561 (15.7%)	
Other	1170 (1.5%)	362 (1.9%)	213 (2.1%)	
White	53,487 (67.4%)	12,860 (66.8%)	6656 (66.7%)	
History hypertension	23,183 (29.2%)	5982 (31.1%)	3587 (36.0%)	< 0.001
History diabetes mellitus	6608 (8.3%)	1949 (10.1%)	1185 (11.9%)	< 0.001
Terminal creatinine	0.94 (0.69–1.46)	0.90 (0.65–1.45)	0.92 (0.66–1.50)	< 0.001
Height (cm)	172.7 (164.0–179.0)	172.0 (164.0–179.0)	171.0 (163.0–178.0)	0.003
Weight (kg)	81.6 (68.5–97.4)	82.4 (69.1–98.9)	82.9 (69.1–99.0)	< 0.001
DCD	23,394 (29.5%)	7091 (36.9%)	4186 (42.0%)	< 0.001
HCV serostatus or NAT‐positive	7980 (10.1%)	2001 (10.4%)	940 (9.4%)	0.03
Pumping at any time	44,601 (56.2%)	12,808 (66.6%)	7263 (72.8%)	< 0.001
Cold ischemia time, hours	18.5 (13.3–23.6)	20.0 (15.9–24.0)	19.8 (16.0–23.8)	< 0.001
Distance from donor to transplant hospital, miles	90 (15–207)	125 (38–212)	128 (41–216)	< 0.001
Sharing				< 0.001
Local	46,323 (58.4%)	7540 (39.2%)	3887 (39.0%)	
Regional	15,737 (19.8%)	4991 (25.9%)	2567 (25.7%)	
National	17,330 (21.8%)	6714 (34.9%)	3522 (35.3%)	
KDPI	39% (20%–60%)	42% (23%–63%)	48% (28%–71%)	< 0.001
Recipient				
Age, yo	55 (44–64)	57 (45–65)	58 (47–66)	< 0.001
Peripheral vascular disease	9958 (12.5%)	2751 (14.3%)	1296 (13.0%)	< 0.001
Prior kidney transplant	8566 (10.8%)	2034 (10.6%)	974 (9.8%)	0.006
Height (cm)	170.2 (162.6–177.8)	170.2 (162.6–177.8)	170.2 (162.6–177.8)	0.19
Weight (kg)	81.2 (68.4–95.5)	81.5 (68.8–95.6)	81.1 (68.4–95.2)	0.30
Diabetes mellitus	30,167 (38.0%)	7778 (40.4%)	4133 (41.4%)	< 0.001
Duration of dialysis				< 0.001
Preemptive	7993 (10.1%)	2483 (12.9%)	1365 (13.7%)	
≤ 1 year	7256 (9.1%)	2004 (10.4%)	1106 (11.1%)	
> 1 and ≤ 3 years	17,855 (22.5%)	5028 (26.1%)	2633 (26.4%)	
> 3 years	46,286 (58.3%)	9730 (50.6%)	4872 (48.8%)	
Renal disease				< 0.001
Glomerular disease	14,689 (18.5%)	3333 (17.3%)	1595 (16.0%)	
Diabetes mellitus	24,353 (30.7%)	6246 (32.5%)	3291 (33.0%)	
Hypertensive	18,554 (23.4%)	4363 (22.7%)	2357 (23.6%)	
Other	16,192 (20.4%)	4109 (21.4%)	2107 (21.1%)	
Polycystic kidney disease	5602 (5.1%)	1194 (6.2%)	626 (6.3%)	
CPRA	0 (0–50)	0.03 (0–55.8)	0.01 (0–54.2)	< 0.001
cPRA sensitivity groups				< 0.001
Low (0%–19%)	52,346 (66.0%)	12,021 (62.5%)	6264 (62.8%)	
Moderate (20%–79%)	13,512 (17.0%)	3692 (19.2%)	1959 (19.6%)	
High (80%–94%)	5266 (6.6%)	1369 (7.1%)	662 (6.6%)	
Very high (95%–100%)	8266 (10.4%)	2163 (11.2%)	1091 (11.0%)	
Transplant factors				
HLA mismatches				< 0.001
0	3907 (4.9%)	930 (4.8%)	508 (5.1%)	
1	987 (1.2%)	248 (1.3%)	104 (1.0%)	
2	3825 (4.8%)	866 (4.5%)	646 (4.7%)	
3	11,454 (14.4%)	2657 (13.8%)	1303 (13.1%)	
4	22,002 (27.7%)	5259 (27.3%)	2672 (26.8%)	
5	25,713 (32.4%)	6303 (32.8%)	3281 (32.9%)	
6	11,502 (14.5%)	2982 (15.5%)	1644 (16.5%)	
Induction immunosuppression				< 0.001
Steroids only	1607 (2.0%)	345 (1.8%)	72 (0.7%)	
Lymphocyte depleting	63,605 (80.1%)	15,805 (82.1%)	8291 (83.1%)	
Nonlymphocyte depleting	10,570 (13.3%)	2377 (12.4%)	1255 (12.6%)	
No induction agent	3608 (4.5%)	718 (3.7%)	358 (3.6%)	

*Note:* DCD, donor after circulatory death.

Abbreviations: HCV, hepatitis C virus; KDPI, Kidney Donor Profile Index; NAT, nucleic acid testing.

^1^1/1/2024–6/30/2024.

^2^Chi‐square test for categorical and Kruskal–Wallis test for continuous variables.

^3^Count and % for categorical and median and IQR for continuous variables.

Consistent with these shifts, the median KDPI increased from 39% (IQR 20%–60%) in 2018–2022 to 48% (IQR 28%–71%) in 2024. Preservation practices also changed: Pumping rose to 72.8% in 2024, while national sharing increased to 35.3%, contributing to longer cold ischemia times and greater travel distances. Recipient characteristics shifted modestly, with a slight increase in median age (55 to 58 years) and a higher prevalence of diabetes mellitus (41.4% in 2024 vs. 38.0% in 2018–2022).

### 3.4. Kaplan–Meier Graft Survival

**FIGURE 3:**
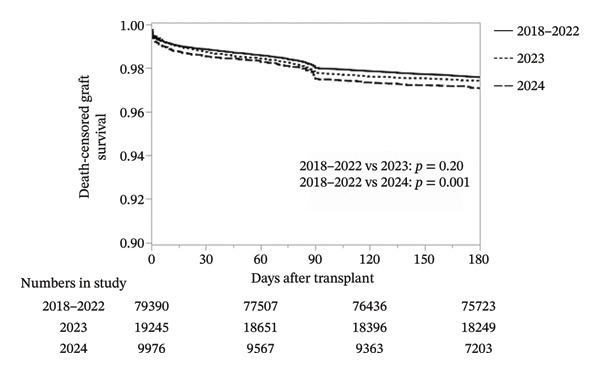
Kaplan–Meier curves for 180‐day death‐censored graft survival by era.

Unadjusted Kaplan–Meier analysis revealed a small but statistically significant decline in 180‐day death‐censored graft survival over time (Figure 3). Survival was 97.6% for transplants performed in 2018–2022, compared with 97.4% in 2023 and 97.0% in 2024 (*p* < 0.001 for trend). Although the absolute difference was modest, separation of survival curves occurred early and persisted throughout follow‐up, consistent with an early warning of outcome decline.

### 3.5. Multivariable Cox Analysis

In unadjusted Cox models, transplant year was associated with higher risk of graft loss in 2024 (HR 1.22, *p* = 0.001), but this association was not significant after adjustment for donor, recipient, and transplant factors (HR 1.08, 95% CI 0.95–1.22, *p* = 0.24) (Table 3). Instead, established donor risk factors explained the decline. Overdose as the mechanism of death was protective (HR 0.73, *p* < 0.001), whereas older donor age (55–64 years: HR 1.43; ≥ 65 years: HR 1.47, both *p* < 0.001), DCD status (HR 1.57, *p* < 0.001), donor hypertension (HR 1.46, *p* < 0.001), and donor diabetes (HR 1.33, *p* < 0.001) were strongly associated with graft failure. Prolonged cold ischemia and national sharing also contributed to worse outcomes, while pumping was modestly protective (HR 0.89, *p* = 0.01).

**TABLE 3:** Multivariable Cox proportional hazards model for 180‐day death‐censored graft failure.

Variables	Univariable			Multivariable		
	HR	95% CI	*p* value	HR	95% CI	*p* value
Year						
2018–2022	Ref					
2023	1.07	0.97–1.18	0.20	1.01	0.91–1.11	0.94
2024	1.22	1.08–1.38	0.001	1.08	0.95–1.22	0.24
Donor						
Single kidney vs en bloc/dual	0.55	0.45–0.68	< 0.001	1.11	0.86–1.43	0.42
Overdose deaths vs other	0.59	0.53–0.67	< 0.001	0.73	0.64–0.83	< 0.001
Age groups						
0–54 yo	Ref					
55–64yo	1.68	1.54–1.84	< 0.001	1.43	1.30–1.58	< 0.001
≥ 65 yo	1.57	1.30–1.89	< 0.001	1.47	1.21–1.79	< 0.001
Donor ethnicity						
Asian	1.09	0.86–1.38	0.48	1.04	0.82–1.32	0.75
Black	1.05	0.94–1.17	0.40	1.04	0.93–1.16	0.48
Hispanic	0.92	0.82–1.03	0.13	0.93	0.83–1.04	0.19
Other	0.75	0.53–1.05	0.10	0.77	0.55–1.09	0.14
White	Ref					
History hypertension	1.73	1.61–1.87	< 0.001	1.46	1.34–1.59	< 0.001
History diabetes mellitus	1.70	1.53–1.89	< 0.001	1.33	1.19–1.49	< 0.001
Terminal creatinine	1.00	0.98–1.03	0.75	1.07	1.04–1.10	< 0.001
Height (cm)	0.99	0.988–0.992	< 0.001	0.984	0.981–0.986	< 0.001
Weight (kg)	1.001	0.999–1.002	0.19	1.002	1.001–1.005	0.002
DCD	1.57	1.46–1.70	< 0.001	1.57	1.45–1.71	< 0.001
HCV serostatus or NAT‐positive	0.71	0.61–0.81	< 0.001	0.90	0.77–1.04	0.16
Pumping at anytime	1.23	1.13–1.33	< 0.001	0.89	0.81–0.97	0.01
Cold ischemia time, hours	1.03	1.02–1.04	< 0.001	1.02	1.01–1.03	< 0.001
Sharing						
Local	Ref					
Regional	1.21	1.10–1.33	< 0.001	1.09	0.98–1.20	0.10
National	1.31	1.21–1.44	< 0.001	1.15	1.04–1.27	0.01
Recipient						
Age groups						
18–35 yrs	Ref					
36–64 yrs	1.23	1.08–1.41	0.002	0.96	0.83–1.10	0.56
≥ 65 yrs	1.49	1.29–1.71	< 0.001	1.04	0.89–1.22	0.63
Peripheral vascular disease	1.27	1.15–1.41	< 0.001	1.16	1.04–1.29	0.01
Prior kidney transplant	1.03	0.91–1.16	0.66	1.19	1.03–1.39	0.02
Height (cm)	1.003	0.99–1.01	0.09	0.991	0.986–0.996	< 0.001
Weight (kg)	1.007	1.006–1.009	< 0.001	1.012	1.009–1.015	< 0.001
Diabetes mellitus	1.17	1.09–1.27	< 0.001	0.93	0.84–1.02	0.13
Duration of dialysis						
Preemptive	Ref					
≤ 1 year	1.00	0.83–1.20	0.99	1.02	0.85–1.23	0.81
> 1 and ≤ 3 years	0.09	0.96–1.29	0.16	1.11	0.95–1.28	0.19
> 3 years	1.28	1.12–1.46	< 0.001	1.38	1.20–1.58	< 0.001
Renal disease						
Glomerular disease	Ref					
Hypertensive	0.94	0.85–1.03	0.17	0.94	0.84–1.05	0.24
Other	1.01	0.91–1.11	0.87	1.14	1.02–1.29	0.03
Polycystic kidney disease	0.83	0.70–0.97	0.02	0.91	0.76–1.08	0.28
cPRA	0.997	0.996–0.998	< 0.001	0.99	0.98–1.01	0.10
Transplant factors						
HLA mismatches						
0–3	Ref					
4–6	1.17	1.07–1.28	< 0.001	1.12	1.02–1.23	0.02
Induction immunosuppression						
Biological agents	Ref					
No induction agent	1.95	1.70–2.24	< 0.001	2.07	1.80–2.39	< 0.001
Steroids only	1.03	0.78–1.35	0.85	1.18	0.89–1.56	0.25

*Note:* DCD, donor after circulatory death.

Abbreviations: HCV, hepatitis C virus; KDPI, Kidney Donor Profile Index; NAT, nucleic acid testing.

Recipient risk factors had more limited influence in the adjusted model. Dialysis duration > 3 years (HR 1.38, *p* < 0.001), prior kidney transplant (HR 1.19, *p* = 0.02), higher body weight (HR 1.012 per kg, *p* < 0.001), and greater HLA mismatch (HR 1.12, *p* = 0.02) were independently associated with graft loss. Induction with no immunosuppressive agent carried the highest risk of graft loss (HR 2.07, *p* < 0.001).

### 3.6. Secondary Outcomes

Other clinically relevant outcomes also declined over the study period (Table 4). The incidence of DGF rose from 31.7% in 2018–2022 to 35.6% in 2024 (*p* < 0.001). PNF increased modestly from 1.1% to 1.5% (*p* < 0.001). Median serum creatinine at six months rose from 1.33 to 1.40 mg/dL (*p* < 0.001). These parallel trends reinforce the conclusion that the evolving donor pool has begun to adversely affect early post‐transplant outcomes.

**TABLE 4:** Secondary outcomes (DGF, PNF, and 6‐month serum creatinine) by era.

	Year			*p* value^2^
	2018–2022	2023	2024^1^	
Delayed graft function	25,132 (31.7%)^3^	6546 (34.0%)	3546 (35.6%)	< 0.001
Primary nonfunction	850 (1.1%)	241 (1.3%)	152 (1.5%)	< 0.001
Serum creatinine at 6 months	1.33 (1.09–1.69)	1.36 (1.10–1.70)	1.40 (1.12–1.79)	< 0.001

^1^1/1/2024–6/30/2024.

^2^Chi‐square test for categorical and Kruskal–Wallis test for continuous variables.

^3^Count and % for categorical and median and IQR from continuous variables.

## 4. Discussion

Over the past decade, the growth in deceased kidney donation in the United States was fueled in large part by younger donors who died from drug overdoses. Despite initial concerns about infection risk, these organs were generally of high quality, and their use was associated with excellent transplant outcomes) [1, 2]. The present analysis shows that this era may be ending. Beginning in 2023, overdose‐death donors declined sharply, and the donor pool has simultaneously shifted toward older donors, higher rates of comorbidities, and increasing reliance on DCD. These cumulative changes have led to a greater proportion of high‐KDPI kidneys, rising nonutilization rates, and measurable declines in unadjusted short‐term graft survival.

Although the absolute decrease in 180‐day graft survival appears modest (97.6% in 2018–2022 vs. 97.0% in 2024), the early separation of Kaplan–Meier curves and the persistence of these differences throughout follow‐up suggest an early indicator that donor quality is beginning to adversely affect outcomes. Importantly, after adjustment for donor, recipient, and transplant factors, transplant year itself was no longer an independent predictor of graft loss. This indicates that the decline in outcomes is not attributable to systemic or era effects, but rather to the underlying transformation of the donor pool.

Given limitations in current OPTN data regarding the capture of normothermic regional perfusion (NRP) utilization, we were unable to directly evaluate the impact of NRP on outcomes, such as DGF and PNF. However, clinical trials have consistently demonstrated favorable effects of NRP on these outcomes [8]. In the context of increasing adoption of NRP in recent years, it is plausible that NRP utilization may partially blunt the adverse outcomes observed in this study.

Our findings are consistent with established evidence regarding individual risk factors. Older donor age, donor hypertension and diabetes, high KDPI, and DCD status are all strongly associated with graft loss [5, 6]. The decline in overdose donors, who were typically younger and healthier, likely removed a source of protective effect, leaving a donor pool increasingly dominated by higher‐risk organs. Secondary outcomes, including increased DGF, higher rates of PNF, and rising serum creatinine at six months, further support the conclusion that organ quality is worsening at a population level.

These shifts raise important clinical and policy implications. First, transplant programs must adapt to a higher‐risk donor pool by refining allocation strategies, optimizing perioperative management, and expanding use of machine perfusion and novel preservation methods [9, 10]. Second, strategies, such as NRP [8], to minimize discard of marginal kidneys while avoiding recipient harm, will be critical as high‐KDPI kidneys constitute a growing share of the pool. Finally, the transplant community must recognize that the gains in organ availability achieved during the peak of the opioid epidemic may not be sustained in the current era, and proactive measures are needed to maintain outcomes as donor risk increases.

This study has several limitations. As a retrospective registry analysis, results are subject to potential misclassification, incomplete reporting, and unmeasured confounders. Follow‐up for 2024 transplants was limited to six months, and longer‐term outcomes may further clarify the extent of risk associated with current donor trends. Nevertheless, the consistency of findings across multiple outcomes—graft survival, DGF, and PNF—strengthens the validity of the observed early decline.

An additional limitation of this study is the changes to KDPI calculation and organ allocation that occurred during the study period. Revisions to KDPI calculation, including the removal of race [11] and hepatitis C virus (HCV) status [12], have resulted in substantial changes in the KDPI assigned to a given donor, which may limit direct comparisons between periods before and after these changes. However, given the magnitude of the observed effect sizes, these differences are unlikely to meaningfully alter the primary findings. From the recipient perspective, contemporaneous changes, including the elimination of race‐based estimated glomerular filtration rate (eGFR) calculation and the potential for shorter time to transplantation [13], may further confound interpretation, particularly in light of the well‐established impact of dialysis vintage on post‐transplant outcomes [14]. Finally, changes to the kidney allocation system itself represent another potential source of temporal variation. Although increased cold ischemia time under the new allocation framework has not been associated with higher rates of DGF to date [15], it may be too early to fully appreciate the long‐term impact of these changes on transplant outcomes.

In conclusion, the U.S. kidney donor pool is undergoing a fundamental transition toward higher‐risk profiles, marked by fewer overdose donors, increasing DCD use, older donor age, and more comorbidities. These changes have already translated into modest but significant declines in short‐term graft outcomes. Given the overall worsening donor profile, further attention is required on novel techniques to reduce kidney nonutilization and improve outcomes, such as NRP and other novel kidney preservation methods [16]. Continued surveillance and proactive adaptation will be essential to sustain the success of kidney transplantation in the years ahead.

**Nomenclature**
KDRI: Kidney Donor Risk Index
UNOS: United Network for Organ Sharing
OPTN: Organ Procurement and Transplantation Network
cPRA: Calculated panel reactive antibody
HLA: Human leukocyte antigen
PVD: Peripheral vascular disease
DGF: Delayed graft function
EPTS: Estimated post‐transplant survival
DCD: Donors after circulatory death

## Funding

No funding was received for this manuscript.

## Conflicts of Interest

The authors declare no conflicts of interest.

## Data Availability

Data for this study were obtained from the OPTN. The UNOS, as the contractor for OPTN, provided access to de‐identified national registry datasets.
